# Supplementary material for: Recombinant TP-84 Bacteriophage Glycosylase–Depolymerase Confers Activity against Thermostable Geobacillus stearothermophilus via Capsule Degradation
Source: Int J Mol Sci. 2024 Jan 5;25(2):722. doi: 10.3390/ijms25020722 (PMC10815759; doi:10.3390/ijms25020722)
Supplement: Supplementary file 1 [file ijms-25-00722-s001.zip › Supplementary File S3.pdf]

Supplementary file S3

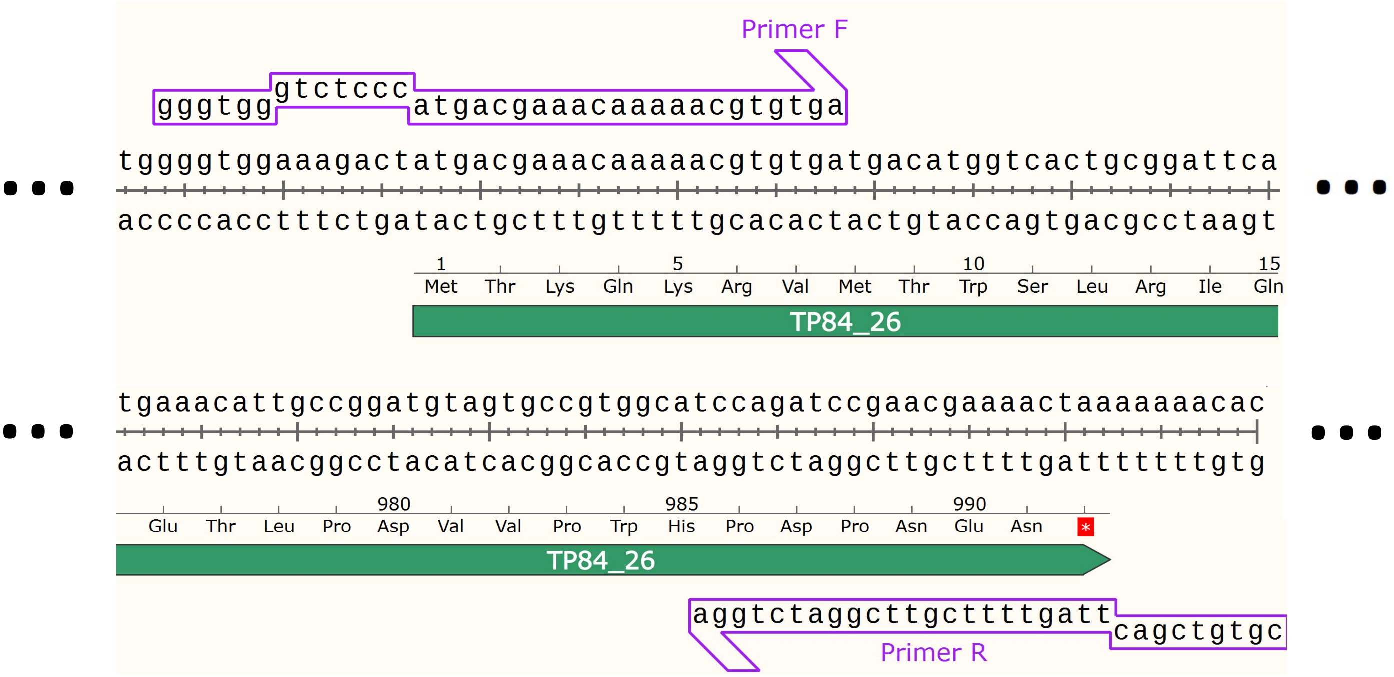

Figure S1. DNA primers used for the PCR amplification of the TP84_26 gene from the TP-84 genome. The figure illustrates the mutagenic PCR primers designed to introduce BsaI and SalI recognition sequences at the respective flanking regions of the TP84_26 gene. The introduced BsaI and SalI sites enable the seamless fusion of the amplified TP84_26 ORF with the start codon of the pET21d vector during the cloning.

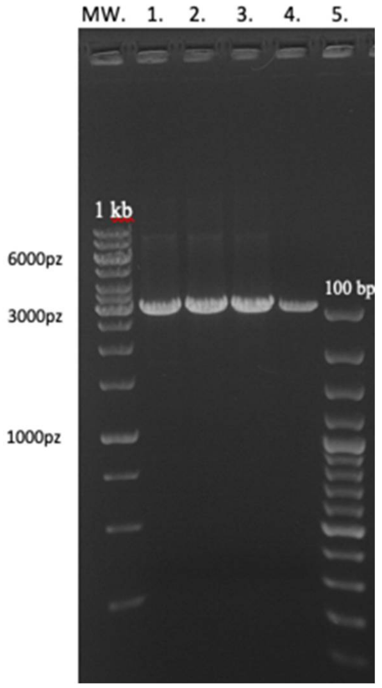

Figure S2. The PCR amplification products of the TP84_26 gene at different annealing temperatures. Lane MW, Thermo Scientific™ O'GeneRuler 1 kb DNA Ladder; lane 1, the TP84_26 amplification product, annealing temperature 49.7°C; lane 2, the TP84_26 amplification product, annealing temperature 57°C; lane 3, the TP84_26 amplification product, annealing temperature 67.1°C; lane 4, the TP84_26 amplification product, annealing temperature 72°C; lane 5, GeneRuler™ 100 bp Plus DNA Ladder.

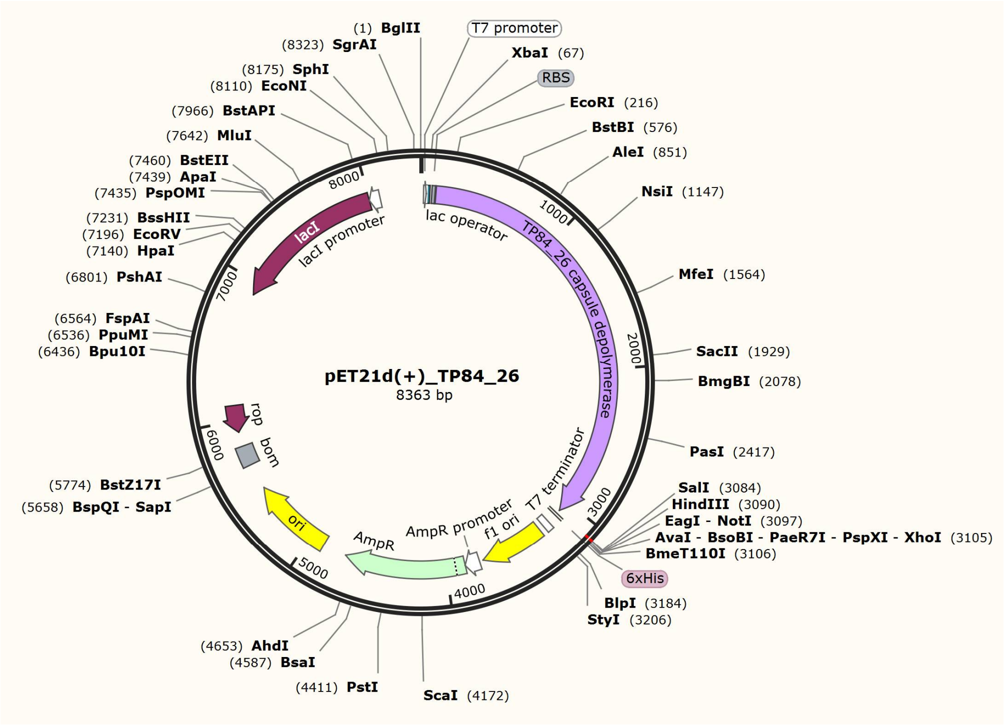

(a)

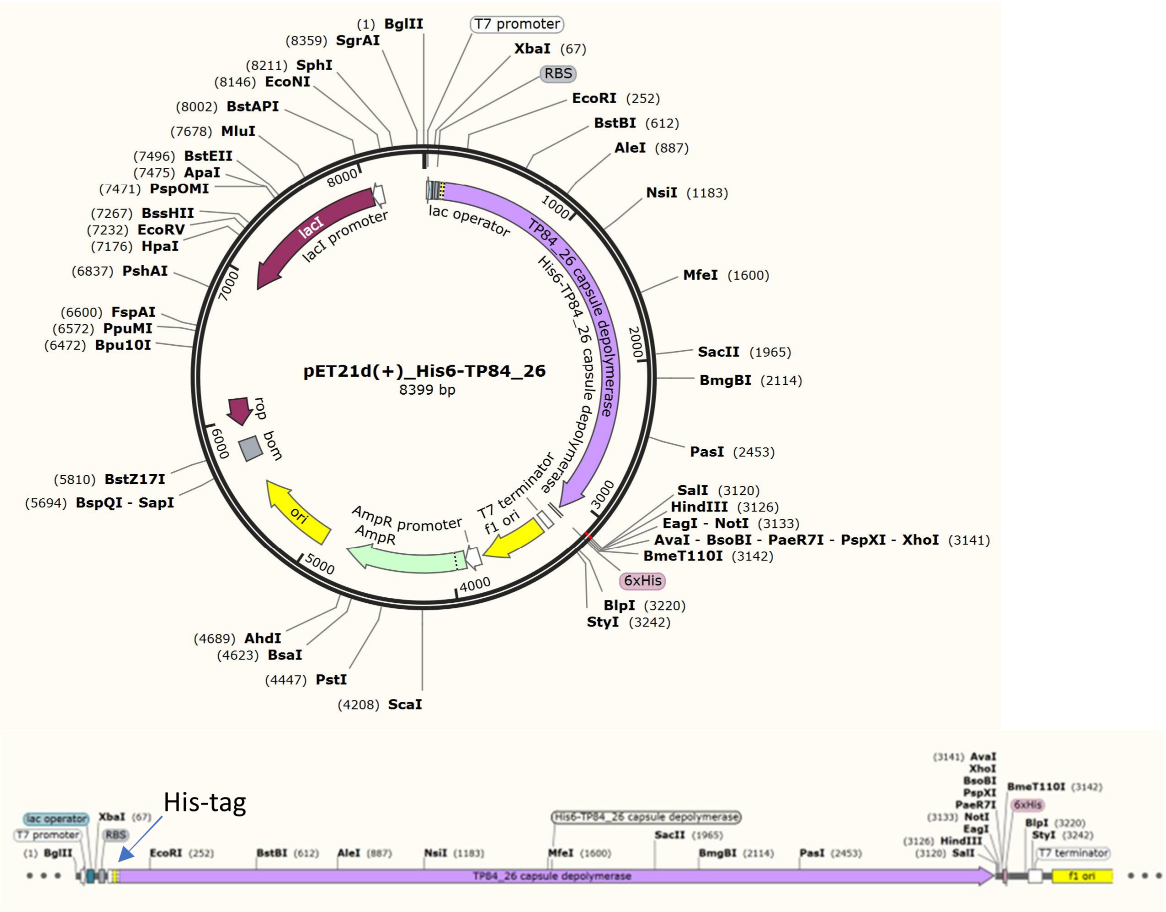

(b)

Figure S3. Genetic maps of the recombinant pET21d(+)_TP84_26 and pET21d(+)_His6-TP84_26 constructs (pET21d(+) backbone, T7-lac promoter, Ribosome Binding Site (RBS), native or His6-TP84_26 ORF, transcription terminator. (a) pET21d(+)_TP84_26. (b) pET21d(+)_His6-TP84_26.

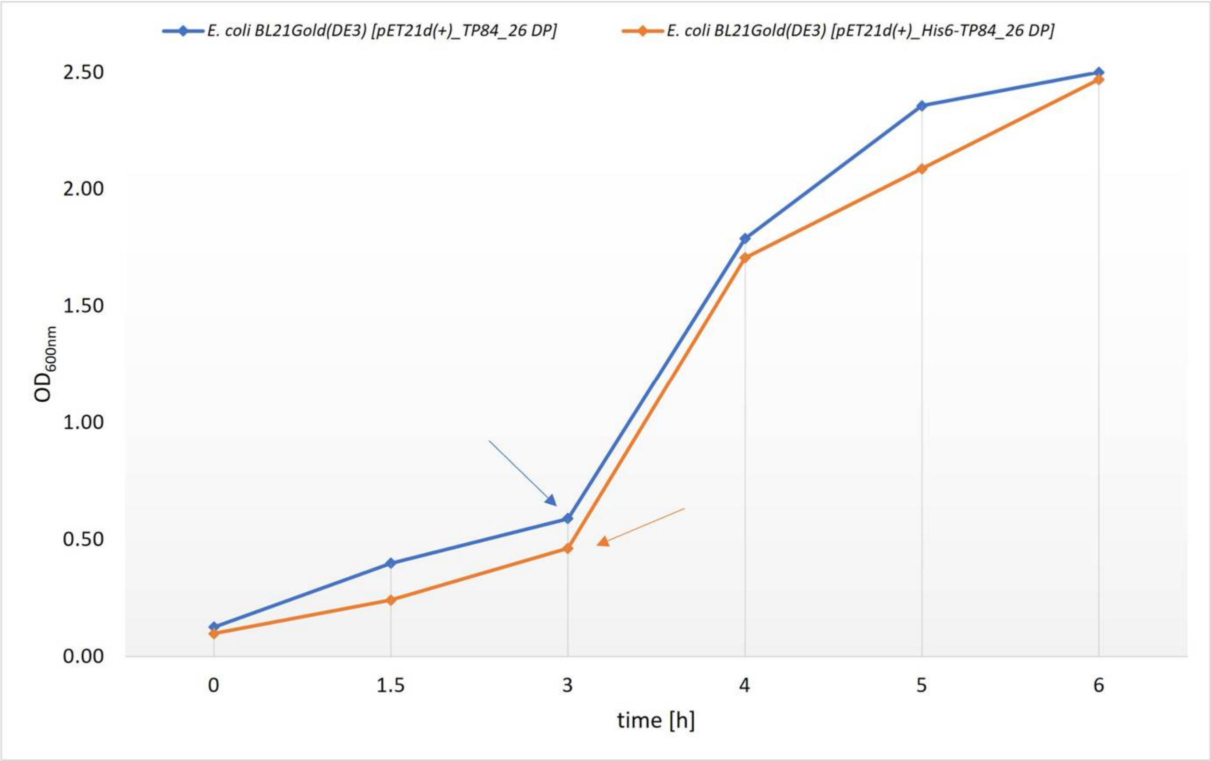

Figure S4. The growth curves for the recombinant *E. coli* BL21-Gold(DE3) [pET21d(+)_His6-TP84_26] and *E. coli* BL21-Gold(DE3) [pET21d(+)_TP84_26] bacterial cultures. The recombinant gene expression was induced with 1 mM IPTG (marked with arrows). The cultures were conducted three hours after induction.

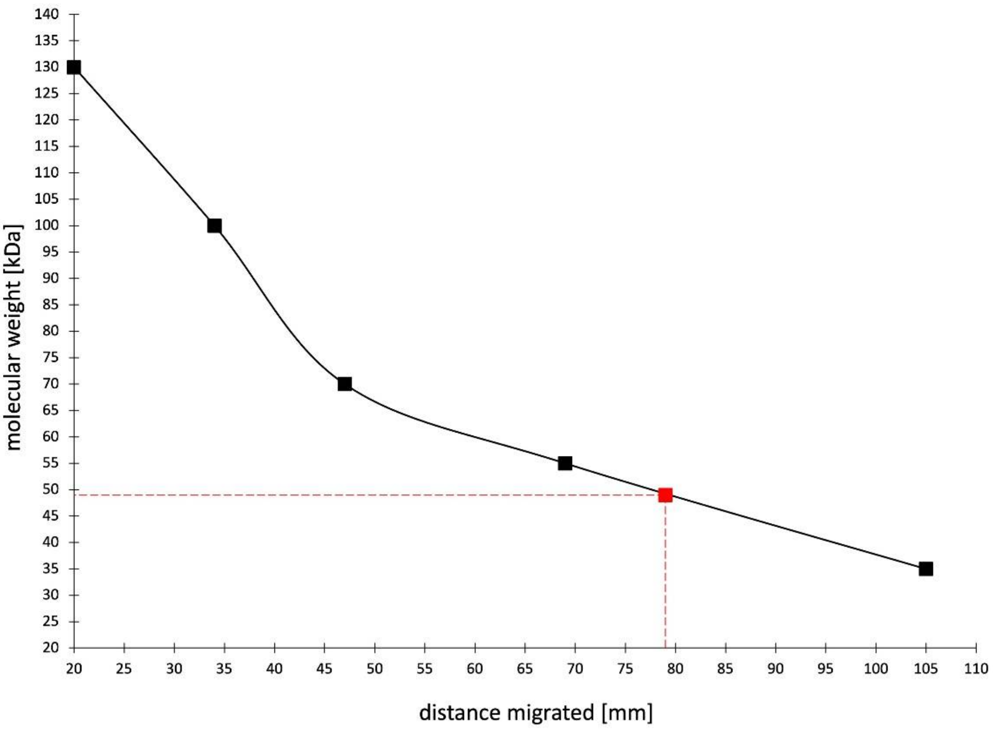

Figure S5. Determination of the molecular weight of a proteolytic TP84_26 depolymerase fragment reacting with anti-TP84 antibodies (Figure 2b, lane 1).

corresponding to Fig. 3, panel d

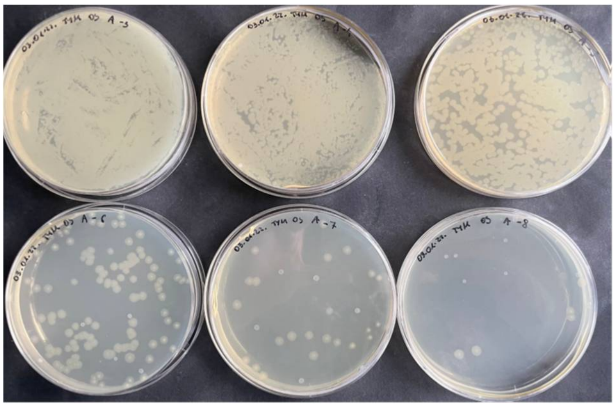

corresponding to Fig. 3, panel e

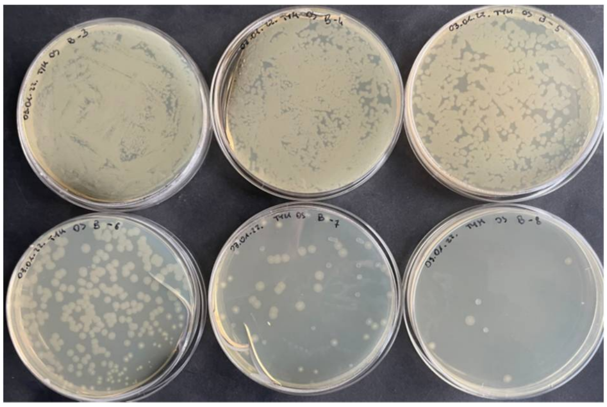

corresponding to Fig. 3, panel f

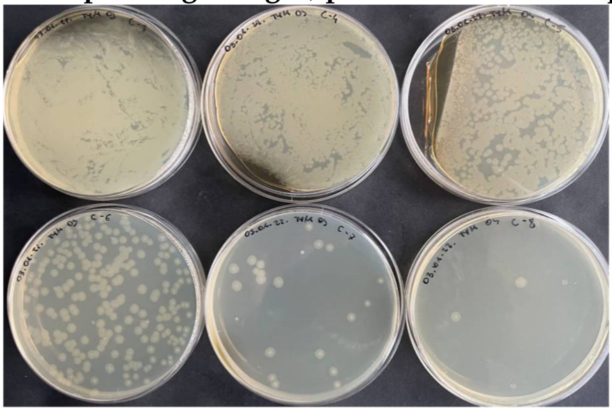

corresponding to Fig. 3, panel g

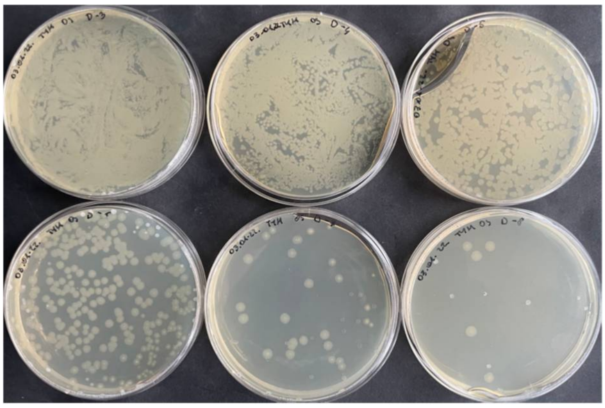

corresponding to Fig. 3, panel h

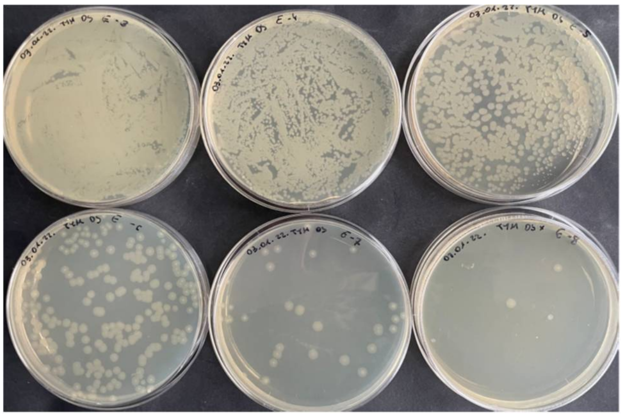

Figure S6. Viability of the *G. stearothermophilus* substrate cells upon exposition to sodium azide/streptomycin and enzymatic capsules removal. The substrate cells (and/or embedded within cells spores) are retaining essentially the same rate of survival upon transient exposition to sodium azide/streptomycin and capsules removal, as determined by bacteria titrations of *G. stearothermophilus* cells, taken from reaction shown in Fig. 3, panels d-h.
